# Total hip arthroplasty: what information do we offer patients on websites of hospitals?

**DOI:** 10.1186/1472-6963-11-83

**Published:** 2011-04-19

**Authors:** Jaap JJ Brunnekreef, Berend W Schreurs

**Affiliations:** 1 Department of Orthopaedics, Radboud University Nijmegen Medical Centre, Nijmegen, the Netherlands

## Abstract

**Background:**

Physicians face a new challenge; the self-educated patient. The internet is an important source that patients use to become self-educated. However, the individual choice for best treatment is difficult. The aim of this study was to investigate what kind of information is offered to total hip arthroplasty patients by internet and what information is appreciated by them.

**Methods:**

Websites of orthopedic departments of all hospitals in the Netherlands were evaluated. In addition, a cohort of 102 patients, diagnosed with arthritic joint disorders, filled in an online survey and gave their opinion concerning the importance of this information.

**Results:**

Eighty different orthopedic websites of hospitals were identified. Websites presented information regarding the orthopedic staff surgeon (76%) and the postoperative rehabilitation process (66%). They also offered referral to other orthopedic websites (61%), the opportunity to make an outpatient appointment (21%), and the opportunity to submit an online question (15%). Patients rated the presence of information regarding prosthesis survival as very important (> 70%). However, the information on the type of prosthesis used by the hospital, and survival data of the prosthesis, were only present in ~ 9% and 5% respectively, of the websites.

**Conclusions:**

The content of health information on websites of hospitals is highly variable for total hip arthroplasty. Information regarding the hip implant and prosthesis survival is highly appreciated by patients, however, mostly absent on orthopedic websites in the Netherlands. The internet provides an enormous potential for orthopedic surgeons to inform the self-educated patient.

## Background

A new challenge physicians are currently facing is the self-educated patient. Today, patients are much better informed on their disease and the condition that affects them than before. These new, self-educated patients are the consequence of cultural changes and improved access to the World Wide Web [1-5]. The internet provides patients with an accessible wisdom of online scientific knowledge and medical information. This trend was observed in 2001, where approximately 40% of internet users reported using it to look for advice or information about health or health care [1].

These self-educated patients can be of real value to physicians, as they are able to have a critical discussion on the suggested choice of treatment. However, the self-educated patient also warrants careful consideration. The quality and content of health information on the internet is highly variable [6]. As a consequence, patients can get confused or misinformed or develop unrealistic expectations about their treatment options [7-11]. In addition, the self-educated patients are also influenced by medical information about hip surgery that is brought to their attention by advertisements. This direct-to-consumer advertising is gaining popularity in orthopedic surgery [7,8]. Therefore, patients should have access to sites that provide them with independent, high quality and realistic information [8,10]. Clearly, there is a role for independent organizations, e.g. universities, general and private hospitals, to provide this realistic online information.

The aim of this study was to investigate what kind of online information is available for patients facing a total hip arthroplasty on websites of hospitals in the Netherlands, and to investigate what information is appreciated by them.

## Methods

### Screening list

In consultation with orthopedic surgeons of our department, we developed a screening list of items that orthopedic surgeons expected to be of special interest to patients facing a total hip arthroplasty. With use of this screening list, the websites of all hospitals in the Netherlands were evaluated on the presence of information related to total hip arthroplasty. The online screening was performed by JB in the period March to April 2009. The websites of 8 Dutch university medical centers, 85 general, and 35 categorical hospitals were screened. Hospitals with multiple locations were registered as one hospital if the website covered all locations. When each location exhibits a separate website, the website was considered as a different website and was screened and analyzed separately.

### Patient's opinion

In association with the Dutch Rheumatic Patients Organization and the Dutch Polyarthrosis Peer Association, the items on our screening list were evaluated. Both patient organizations invited a panel of members. All involved panel members suffered from arthritic joint disorders. Panel members received an electronic invitation in which they were asked to fill in an online survey. The online survey consisted of questions that measured the importance of the topics on the screening list. The importance of information was rated on a five point scale, with score-1; '*very unimportant information' *and score-5; '*very important information'*. At the end of the survey, in an open question, patients were asked whether they had suggestions for additional topics that they felt important to be informed about. All the survey data was obtained anonymous by an independent researcher and was analyzed using descriptive statistics.

## Results

### Information on websites

In the Netherlands, all hospitals have a website available. Because several hospitals displayed internet-sites that covered multiple locations, a total of 97 different websites of hospitals were located. Of these 97 websites, 80 websites had a link to an orthopedic website or a link to a websites of orthopedic partnership. Seventeen hospitals who did not have an orthopedic department, or did not provide orthopedic services, were excluded.

Seventy-six percent of the 80 orthopedic websites presented information about the orthopedic surgeons that worked at the hospital. This information comprised a photograph, the name of the surgeon, and, in some cases, his or her sub-specializations. Information regarding the rehabilitation process was present on 66 percent of orthopedic websites. This information could be retrieved from an electronic information brochure, available on 46 percent of websites, or was described on the website in detail. Sixty-one percent of the orthopedic websites offered referral to other orthopedic websites. These links gave access to minimally two other websites with orthopedic-related information. The opportunity to arrange an online consult was provided by 15 percent of orthopedic websites. This information comprised the opportunity to send an online question to one of the orthopedic surgeons. Twenty-one percent of websites offered the opportunity to make an outpatient appointment by internet (table 1).

**Table 1:** Presence of general information on total hip arthroplasty

Information about;	N	%
Orthopedic surgeon?	61	76,2
Rehabilitation process?	53	66,2
Referral to other orthopedic websites?	49	61,2
Brochure on hip arthroplasty?	37	46,2
Opportunity to make appointment online?	17	21,2
Opportunity to ask question online?	12	15,0

Forty percent of the 80 orthopedic websites presented general information on total hip arthroplasty. We divided information on hip prosthesis into four groups; hip prosthesis fixed with bone cement, prosthesis inserted without bone cement, prosthesis of the hybrid type (in which one part is cemented and the other part not), and information on a special type of hip prosthesis, the so-called resurfacing hip. Detailed information with respect to cemented total hip arthroplasty was present on 21 percent of the websites, 21 percent presented information on resurfacing prosthesis, 19 percent on uncemented prosthesis, and 3 percent on hybrid hip prosthesis. This information comprised an image of the prosthesis and a short description of the operation. Nine percent of the orthopedic websites mentioned what type of implant; cemented, uncemented, resurfacing or hybrid prosthesis was preferably implanted by the hospital or partnership of orthopedic surgeons. One website presented specific information on which type of stem and cup their centre would like to implant. Four websites (five percent) provided specific information on the expected survival of their implants. These websites cited own results or referred to published data elsewhere. One website presented not only the survival of their prosthesis, but they also referred to survival data from literature of other prosthesis not implanted by them (table 2).

**Table 2:** Presence of specific information on total hip arthroplasty

Information about;	N	%
Cemented hip prosthesis?	17	21,2
Resurfacing hip prosthesis?	17	21,2
Uncemented hip prosthesis?	15	18,8
Hybrid hip prosthesis?	2	2,5
Type of prosthesis implanted by department?	7	8,8
Survival of implanted hip prosthesis?	4	5,0
Survival of other total hip prosthesis?	1	1,2

### Online survey

The online survey, regarding the importance of the topics on the screening list, was completed by 102 patients of the Dutch Rheumatic Patients Organization (n = 21) and the Dutch Polyarthrosis Peer Association (n = 81). All items on the screening list, except the opportunity to make an online appointment, were rated by 80 percent of the patients as important to very important information. The most important aspect patients preferred to be informed about was the survival rate of the actual implanted prosthesis. All participating patients would like to be informed about this topic by hospital websites, and 71 percent of patients rated this information as *very important *(see table 3). Additional topics that patients suggested they wished to be informed about are displayed in table 4.

**Table 3:** Patients opinion regarding the importance of information

*'How important do you find this information?'*	very unimportant	unimportant	neutral	important	very important
Information about orthopedic surgeon?	2.9% (3)	2.0% (2)	13.7% (14)	**44.1% (46)**	36.3% (37)
Information about rehabilitation process?	2.0% (2)	0.0% (0)	1.0% (1)	34.3% (35)	**62.7% (64)**
Referral to other orthopedic website?	2.0% (2)	2.0% (2)	14.8% (15)	**56.4% (57)**	24.7% (25)
Brochure on total hip arthroplasty?	2.0% (2)	0.0% (0)	4.0% (4)	24.7% (25)	**69.3% (70)**
Opportunity to make appointment online?	2.0% (2)	4.9% (5)	29.4% (30)	**47.1% (48)**	16.7% (17)
Opportunity to ask question online?	2.0% (2)	1.0% (1)	17.0% (17)	**48.0% (48)**	32.0% (32)
Information about cemented, uncemented, resurfacing, or hybrid hip prosthesis?	1.0% (1)	0.0% (0)	1.0% (1)	28.7% (29)	**69.3% (70)**
Information about type of hip implant?	1.0% (1)	0.0% (0)	2.0% (2)	34.3% (35)	**62.7% (64)**
Information about survival of hip implant?	1.0% (1)	0.0% (0)	0.0% (0)	28.4% (29)	**70.6% (72)**
Information about survival of other hip implants?	1.0% (1)	1.0% (1)	8.9% (9)	37.6% (38)	**51.5% (52)**

**Table 4:** Other topics patients like to be informed about

*'Other topics you would like to be informed about?'*	N
Waiting list	7
Pain management	4
Complications	4
Able to perform in sports	3
Number of operations performed yearly	2
Luxury of hospital room	2
Duration of hospital stay	1

## Discussion

This study investigated the extent of total hip arthroplasty related information available on orthopedic websites of hospitals in the Netherlands. The content of health information on websites of hospitals is highly variable for total hip arthroplasty. Information regarding the hip implant and prosthesis survival is highly appreciated by patients, however, mostly absent on orthopedic websites in the Netherlands. Orthopedic websites evaluated were better at providing information on the rehabilitation process, the orthopedic surgeon, and offering referral to other orthopedic websites, as evident from more than 60 percent of the websites.

Our report is based on a complete review of all hospital websites in the Netherlands. The review focuses on a type of surgery that is done frequently in the Netherlands (more 25.000 hip implants a year). With more than one million operations performed each year worldwide, total hip arthroplasty is considered one of the most successful procedures in modern orthopedic surgery [12]. Total hip replacement is elective surgery, this means that it does not need to be done urgently. Therefore, patients have time and the opportunity to search the internet for medical information to become self-educated about the best treatment option in their specific situation.

### Direct-to-consumer advertising

More recently, orthopedic surgeons, hospitals, and orthopedic device manufacturers have started to advertise their products and services directly to end-users [7,8]. Direct-to-consumer advertisements in newspapers, on television, or the internet has been successfully used by the pharmaceutical industry for many years [11,13,14]. However, misleading information by direct-to-consumer advertising has a negative influence on the doctor-patient relationship in orthopedic surgery [8]. Patients can get confused, misinformed or develop unrealistic expectations about the appropriate treatment option for their condition. To counterbalance this direct-to-consumer advertising, realistic online information about orthopedic products, services, and treatment options on hospital websites is likely to be of vital importance to patients.

There is no doubt that online information plays an eminent role in the propagation of health related information. Results from the National Trends Survey (2003) showed that 63.7% of the US adult population looked for health related information for themselves or relatives at least once in the previous 12 months and half of the population reported searching online first before talking to their physicians [3]. With more than 80 percent of the households connected to the World Wide Web, the Netherlands has one of the highest internet penetrations of Europe [15]. The number of patients that consult the internet for health-related information for themselves and their relatives is presumably even higher.

However, research on choice behavior of patients has shown that offering relevant information alone is not enough to influence the choice of consumers dramatically [16-18]. Other factors, such as the reputation of the physician and oral information from acquaintances (who have already undergone the operation) are important factors that contemporary influence the choice behavior of patients.

### Clinical indicator of performance

The most remarkable finding of our study was that the survival data of the used prosthesis was in general not available on websites of hospitals in the Netherlands. At the moment, the survival of the hip implant is not a clinical indicator of performance in orthopedic total hip surgery in the Netherlands. Orthopedic departments are not required to report this information to national health authorities. Available data on infection parameters, decubitus ulcers, and hospital readmissions are apparently considered more important by policymakers. In contrast, the reported long-term survival of a hip prosthesis can be an important indicator of clinical performance of a department, as satisfying long-term prosthesis survival is what it is all about.

### Limitations

Our study does not investigate additional information on total hip arthroplasty that is provided to out-patients by orthopedic surgeons during a hospital visit. In clinical practices, patients may receive more oral and detailed written information about the type of prosthesis, the surgical procedure, and implant survival during their visit.

According to our online survey, patients do not only want to be informed about the operational technique, type of hip implant or rehabilitation process, but also the length of the waiting list, pain management and complications of having surgery performed (table 4). In our evaluation of websites, however, we did not extract this information from websites. The suggested topics should be considered in future evaluations.

The generalization of our findings may be limited, because the evaluation was done only on websites of hospitals in the Netherlands. Therefore, our conclusions do only apply to situations comparable to the Netherlands.

## Conclusions

Online information of good quality is crucial for patients to become self-educated. The content of health information on websites of hospitals is highly variable for total hip arthroplasty. Orthopedic surgeons have the opportunity to improve the information provided on total hip arthroplasty via the internet. Information on prosthesis survival is important and highly appreciated by patients, however, this information is mostly absent on orthopedic websites in the Netherlands.

## Competing interests

The authors declare that they have no competing interests.

## Authors' contributions

JB performed the online screening of websites, participated in the analysis and interpretation of the data, and drafted the manuscript. BS conceptualized the study, assisted with data interpretation, provided feedback throughout the drafting of this article, and has read and approved the final manuscript.

## Pre-publication history

The pre-publication history for this paper can be accessed here:

http://www.biomedcentral.com/1472-6963/11/83/prepub
